# Dietary vitamin K intake in relation to skeletal muscle mass and strength among adults: a cross-sectional study based on NHANES

**DOI:** 10.3389/fnut.2024.1378853

**Published:** 2024-08-30

**Authors:** Qiong Wang, Pei-pei Chen, Jia-yu Guo, Shi-jia Wang, Yuan-yuan Bao, Yu Zhang, Kang Yu

**Affiliations:** ^1^Department of Clinical Nutrition, Peking Union Medical College Hospital, Peking Union Medical College, Chinese Academy of Medical Sciences, Beijing, China; ^2^Department of Clinical Nutrition, The Second Affiliated Hospital of Soochow University, Suzhou, China

**Keywords:** sarcopenia, vitamin K, NHANES, handgrip strength, skeletal muscle mass

## Abstract

**Background:**

Previous studies revealed that vitamin K might help maintain muscle homeostasis, but this association has received little attention. We aimed to explore the associations of vitamin K intake with skeletal muscle mass and strength.

**Methods:**

We included cross-sectional data from the U.S. National Health and Nutrition Examination Survey (NHANES, 2011–2018). Vitamin K intake was assessed via 24-h recall. Covariate-adjusted multiple linear regression and restricted cubic splines were used to evaluate the associations of dietary vitamin K intake with skeletal muscle mass and strength, measured by dual-energy X-ray absorptiometry and handgrip dynamometer, respectively.

**Results:**

Dietary vitamin K intake was positively associated with skeletal muscle mass in males (β = 0.05747, *p* = 0.0204) but not in females. We also revealed a positive association between dietary vitamin K intake and handgrip strength within the range of 0–59.871 μg/d (*P*_nonlinear_ = 0.049). However, beyond this threshold, increasing vitamin K intake did not cause additional handgrip strength improvements.

**Conclusion:**

We provided evidence for a positive relationship between dietary vitamin K intake and skeletal muscle mass in males. Moreover, our study revealed a nonlinear relationship between dietary vitamin K intake and handgrip strength, highlighting an optimal intake range.

## 1 Introduction

Skeletal muscle comprises approximately 40% of the total body mass and is the main component of lean body mass (LBM) (1), playing a crucial role in maintaining physical condition and mobility (2). In addition to having crucial functions in various physiological processes (3, 4), such as the immune response, glucose regulation, and protein synthesis, it functions as an endocrine organ, secreting myokines that regulate inflammation and other tissues (1, 3). The maintenance of skeletal muscle is fundamental to locomotion, energy homeostasis, and overall quality of life (4–6).

Sarcopenia (7–9), characterized as a progressive and generalized disorder affecting skeletal muscles, has garnered significant attention worldwide because of its association with various adverse outcomes, such as falls, functional decline, frailty, and mortality (8–12). The assessment of handgrip strength and skeletal muscle mass has proven to be a reliable approach for diagnosing sarcopenia (9, 10, 13). These measurements can have predictive value for clinical outcomes, such as identifying individuals at greater risk of prolonged hospitalization durations (5, 11), accelerated functional decline (12), diminished health-related quality of life (13), and increased mortality (6, 12). A multitude of factors contribute to the progressive reduction in skeletal muscle mass and strength, including the natural aging process, pathological conditions, physical inactivity, sedentary behaviors, and inadequate nutritional intake (7, 14). Among the modifiable risk factors, diet plays an increasingly greater role (15, 16). Specifically, protein provide essential amino acids for muscle maintenance and repair (17–19), vitamin D plays a role in muscle function (20, 21), omega-3 fatty acids have anti-inflammatory properties that are important for muscle health (22), and magnesium is involved in muscle contraction (21), all of which notably influence muscle mass and strength.

Several studies have provided evidence suggesting the potential role of vitamin K in regulating energy metabolism within skeletal muscle (23, 24). Cross-sectional studies and randomized controlled trial have highlighted a positive association between high plasma levels of vitamin K and increased muscle strength, increased muscle mass, and superior physical performance (25, 26). Nevertheless, conflicting results have arisen from numerous clinical investigations elucidating the role of vitamin K in maintaining muscle homeostasis (27, 28). Although the specific assessment of dietary vitamin K intake was not undertaken in these studies, they contribute to the notion that vitamin K may exert a significant influence on muscle function.

The present study aims to investigate the association between the dietary intake of vitamin K and both skeletal muscle mass and muscle strength using the National Health and Nutrition Examination Survey (NHANES) data from 2011 to 2018 in the United States (U.S.). Our hypothesis is that dietary vitamin K intake is related to both skeletal muscle mass and muscle strength, and may exert a significant influence on muscle function.

## 2 Materials and methods

### 2.1 Study design

All the data in this analysis were selected from the NHANES, a series of cross-sectional surveys conducted by the Centers for Disease Control and Prevention (CDC). The NHANES surveys adopted a stratified, multistage probability cluster sampling design to select representative samples from United States civilians. These surveys are conducted on a continuous basis in two-year cycles, with selected individuals being invited to participate in a comprehensive interview conducted at their homes, followed by a thorough health examination at mobile examination centers (MECs) to assess their overall health and nutritional status. It is a continuous public database, with nearly 5,000 people accessed yearly. All subjects included in the study provided written informed consent. The survey data and methodological details about the NHANES are available at www.cdc.gov/nchs/nhanes/.

### 2.2 Study participants

For the examination of the association between vitamin K intake and skeletal muscle mass, we incorporated data from all four survey cycles (NHANES 2011–2018). For the analysis of the association between vitamin K intake and muscle strength, we utilized two survey cycles (NHANES 2011–2014) because information on handgrip strength measurements was only available in those two cycles. To ensure the validity and reliability of our results, certain exclusions were made. Participants were excluded if they had missing information on key variables such as dietary vitamin K intake, skeletal muscle mass or handgrip strength measurements or if their reported data exhibited inconsistencies or errors. Additionally, individuals younger than 18 and older than 59 years of age were also excluded from the analysis. As a result of these exclusion criteria, the final dataset for the analysis consisted of 11,189 participants for the assessment of the relationship between vitamin K intake and skeletal muscle mass (Supplementary Figure S1) and 6,892 participants for the evaluation of the association between vitamin K intake and muscle strength (Supplementary Figure S2).

### 2.3 Skeletal muscle mass

In this study, body composition measurements were obtained using whole-body dual-energy X-ray absorptiometry (DXA) technology (Hologic, Inc., Bedford, Massachusetts) (29). To ensure accurate and reliable measurements, individuals with a height exceeding 192.5 cm or a weight exceeding 136.4 kg were excluded from the DXA assessment. Prior to the scan, all metallic objects, except false teeth and hearing aids, were removed to prevent interference with the measurements. The DXA examinations were administered by trained and certified radiology technologists. Further details of the DXA examination protocol are documented in the Body Composition Procedures Manual located on the NHANES website. Appendicular skeletal muscle mass (ASM) was calculated as the sum of lean soft tissue from the limbs (30). ASM is a well-established proxy for assessing skeletal muscle mass (31). Additionally, the appendicular skeletal muscle index (ASMI) was used to quantify skeletal muscle mass, which was calculated as ASMI = ASM (kg)/height (m^2^) (7, 14).

### 2.4 Muscle strength

In this study, handgrip strength was utilized as a proxy indicator of muscle strength (32). For the survey cycles conducted in 2011–2012 and 2013–2014, muscle strength was measured using a handgrip dynamometer (Takei Digital Grip Strength Dynamometer, Model T.K.K.5401). Each hand was tested three times, alternating hands with a 60-s rest between measurements on the same hand. The combined handgrip strength is in kilograms and was calculated as the sum of the largest readings for each hand. Detailed descriptions of the protocol are provided in the NHANES Muscle Strength/Grip Test Procedure Manual available on the NHANES website.

### 2.5 Dietary intake

In NHANES, each participant’s food and nutrient intake was recorded through the 24-h dietary recall interview process, which included two rounds (33). The first round was performed in person, while the second round was completed via telephone between 3 and 10 days later. Notably, not all participants who underwent the telephone interview in the second round dietary recall interview received uniform training in food measurement, which could have introduced variation in the quality of the data recorded. Therefore, our study defined the dietary vitamin K intake (from both food and supplements) of the first round 24-h dietary recall interview as the independent variable. Participants were categorized into four groups (Q1, Q2, Q3, and Q4) based on their dietary vitamin K intake levels. Vitamin K intake from each food or supplement was calculated using the US Department of Agriculture’s Food and Nutrient Database for Dietary Studies (FNDDS), which specifies the nutrient content of various foods and supplements (34).

### 2.6 Covariates

In this study, several important covariates were selected to assess the influence of potential confounding factors on the relationship between dietary vitamin K intake and outcome variables. These covariates were chosen based on previous studies (35, 36), and covariates considered to have multicollinearity issues were manually removed. Eventually, the covariates included in our study of dietary vitamin K intake and skeletal muscle mass are demographic data (age, gender, ethnicity, marital status, body mass index (BMI), smoking status, education), health-related factors (self-reported cancer, hypertension, high cholesterol), laboratory data (total cholesterol, high-density lipoprotein cholesterol (HDL) and glycated hemoglobin) and nutrient intake situation (total vitamin D intake, total Ca intake, and total Mg intake). The covariates included in study of dietary vitamin K intake and muscle strength are demographic data (age, gender, marital status, body mass index (BMI), smoking status), health-related factors (self-reported cancer, hypertension, high cholesterol), laboratory data (total cholesterol, high-density lipoprotein cholesterol (HDL) and glycated hemoglobin) and nutrient intake situation (total vitamin D intake and total Ca intake). Due to a considerable number of missing values, other potential confounding factors were not included in the analysis. All covariates were assumed to confound the relationship between dietary vitamin K intake and outcome variables. By focusing on covariates with minimal missing values, we aimed to mitigate bias and ensure the robustness of the findings.

### 2.7 Statistical analyses

Our study incorporated recommended sampling weights from NHANES to account for planned oversampling and the complex hierarchical, multistage, clustered sampling design. We noticed a skewed distribution among the participants regarding the data on dietary vitamin K intake, so we performed a log transformation. We applied the Box-Cox transformation to outcome variables to meet the assumptions of linear regression. We utilized both simple and multiple linear regression analyses to investigate the relationships between dietary vitamin K intake and the outcome variables. We performed VIF tests to identify potential issues of multicollinearity. If the VIF value is generally greater than 10, it is considered to have severe collinearity (37). Covariates considered with very high correlations were manually removed during subsequent model construction. The crude model did not involve any adjustments for covariates. In Model 1, we adjusted for sociodemographic and behavioral variables, including sex, age, ethnicity, education, marital status, BMI, and smoking status. Model 2 incorporated additional adjustments for all relevant covariates. Furthermore, we employed restricted cubic splines to depict the dose–response relationship between vitamin K intake and the outcome variables. We also conducted subgroup analyses. All statistical analyses were conducted using RStudio v.1.4.1717. We used two-sided tests, and statistical significance was indicated by a *p* value less than 0.05.

## 3 Results

### 3.1 Characteristics of the participants

**Figure 1:**
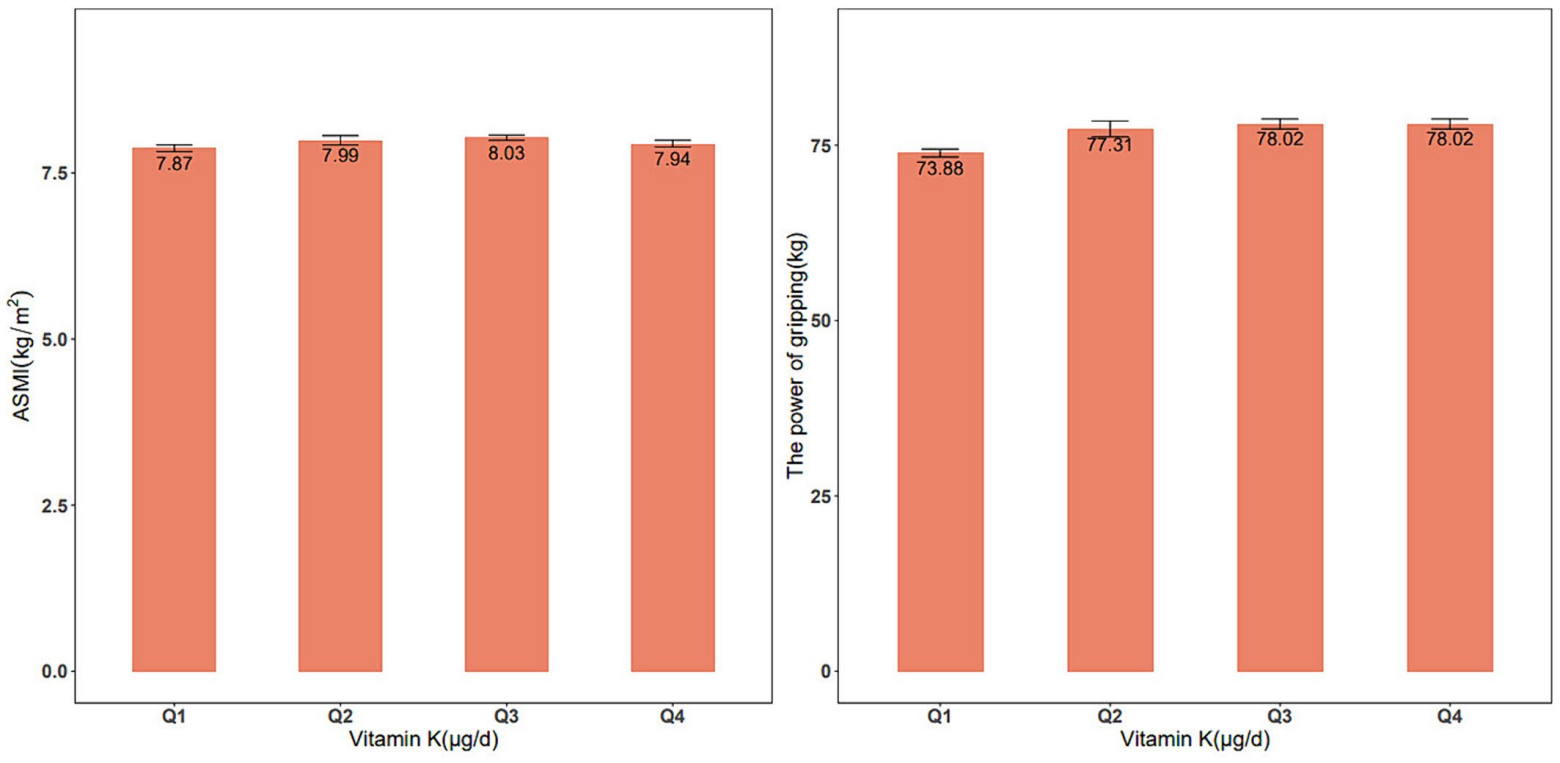
ASMI **(A)** and handgrip strength **(B)** values between the quartiles of vitamin K intake.

From 2011–2018, our analysis included 11,189 eligible participants (50.4% males, n = 5,550) to explore the association between dietary vitamin K intake and skeletal muscle mass. Significant differences in characteristics, such as age, sex, race, BMI, smoking status, education level, marital status, and high cholesterol, were observed among these groups. Furthermore, there were significant differences in dietary protein, Ca, Mg, and vitamin D intake. For the analysis of the association between vitamin K intake and muscle strength during 2011–2014, a total of 6,892 participants were included (50.5% male, *n* = 3,404). Similar trends were observed in the differences among the various dietary vitamin K intake groups. These findings are summarized in Supplementary Tables S3, S4. Figure 1 shows the ASMI and handgrip strength values between the quartiles of vitamin K intake.

### 3.2 Association of dietary vitamin K intake with ASMI

Our analysis employed simple and multiple linear regression models to investigate the association between dietary vitamin K intake and ASMI. Model 1, which adjusted for sociodemographic and behavioral variables, revealed a significant positive association between higher vitamin K intake and greater ASMI (β = 0.011, *p* < 0.001). Nonetheless, this association was no longer present in Model 2, which accounted for all covariates. Due to collinearity issues with protein and total energy in Model 2, these variables were not incorporated into the final model (Supplementary Table S1). The restricted cubic spline analysis was also nonsignificant (*P* for nonlinearity = 0.1512). Subsequent subgroup analyses based on sex, utilizing Model 2 as the final model, indicated a significant positive linear association between dietary vitamin K intake and ASMI in males (β = 0.008, *p* = 0.024). However, this association was not observed in females. Detailed results can be found in Table 1.

**Table 1:** Association of dietary vitamin K intake with ASMI and handgrip strength.

Outcomes	Log Vitamin K intake (μg/d)					
	Crude		Model 1^1^		Model 2^2^	
	β(95%CI)	*P*	β(95%CI)	*P*	β(95%CI)	*P*
ASMI (kg/m^2^)	0.002 (−0.005,0.009)	0.553	0.011 (0.005,0.016)	<0.001	0.005 (−0.001,0.011)	0.07
Handgrip strength (kg)	0.07 (0.026,0.115)	0.002	0.011 (−0.041,0.062)	0.661	−0.012 (−0.071,0.047)	0.665
	Male					
	β(95%CI)	P	β(95%CI)	P	β(95%CI)	P
ASMI (kg/m^2^)	0.003 (−0.005,0.0110)	0.424	0.010969 (0.005,0.017)	0.001	0.008 (0.001,0.014)	0.024
Handgrip strength (kg)	0.013 (−0.028,0.054)	0.522	0.023 (−0.060,0.106)	0.569	−0.015 (−0.116,0.082)	0.753
	Female					
	β(95%CI)	P	β(95%CI)	P	β(95%CI)	P
ASMI (kg/m^2^)	−0.006 (−0.013,0.002)	0.118	0.010 (−0.001,0.021)	0.068	0.004 (−0.008,0.015)	0.522
Handgrip strength (kg)	0.046 (0.010,0.083)	0.015	0.002 (−0.066,0.071)	0.943	−0.003 (−0.070,0.064)	0.930

1 Covariates included in the study are demographic data (age, gender, ethnicity, marital status, body mass index (BMI), smoking status, education).

2 Covariates included in the study of dietary vitamin K intake and ASMI are demographic data (age, gender, ethnicity, marital status, body mass index (BMI), smoking status, education), health-related factors (self-reported cancer, hypertension, high cholesterol), laboratory data (total cholesterol, high-density lipoprotein cholesterol (HDL) and glycated hemoglobin) and nutrient intake situation (total vitamin D intake, total Ca intake, and total Mg intake). Covariates included in study of dietary vitamin K intake and handgrip strength are demographic data (age, gender, marital status, body mass index (BMI), smoking status), health-related factors (self-reported cancer, hypertension, high cholesterol), laboratory data (total cholesterol, high-density lipoprotein cholesterol (HDL) and glycated hemoglobin) and nutrient intake situation (total vitamin D intake and total Ca intake).

### 3.3 Association of dietary vitamin K intake with handgrip strength

**Figure 2:**
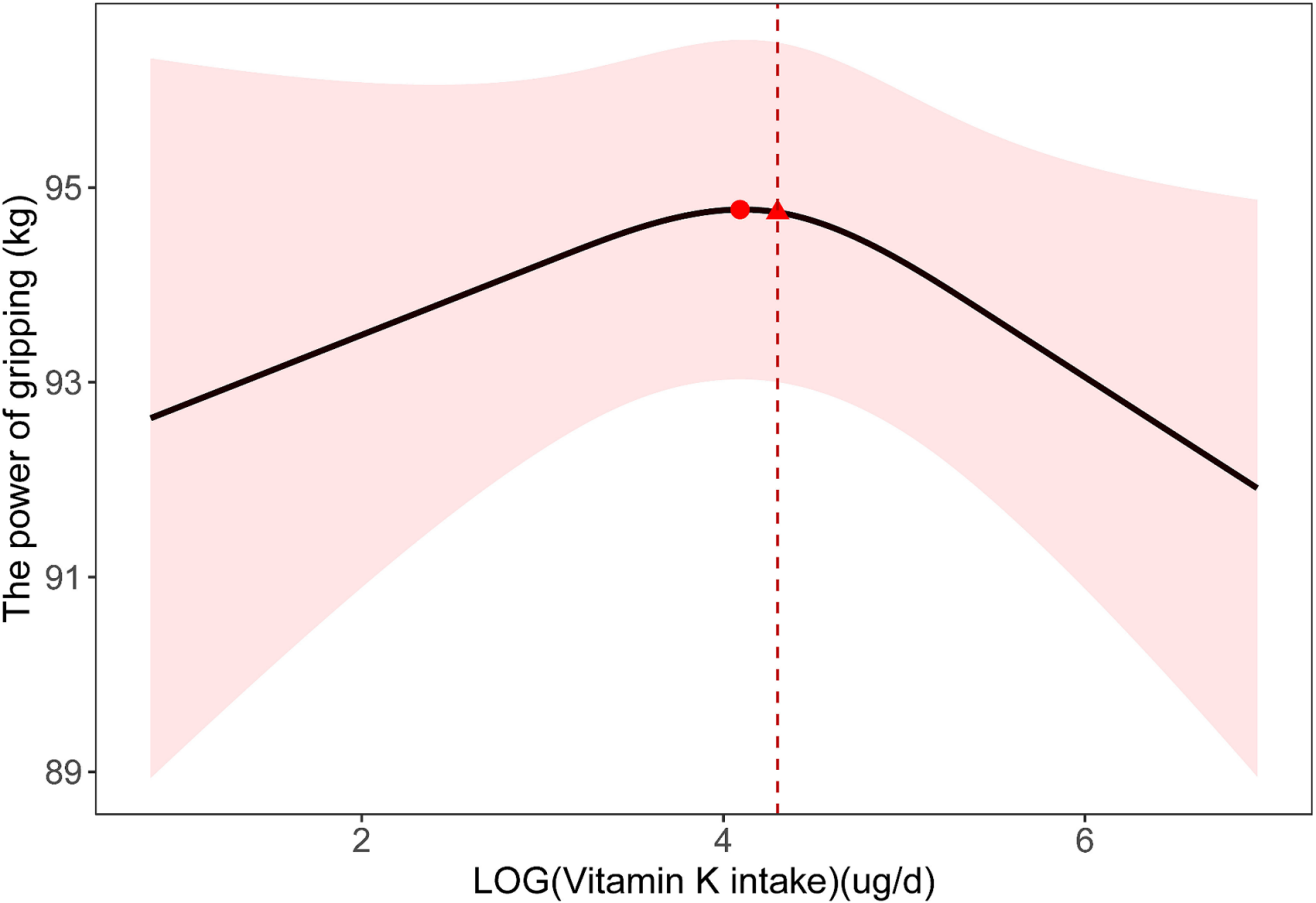
Nonlinear relationship between dietary vitamin K intake and handgrip strength.

**Figure 3:**
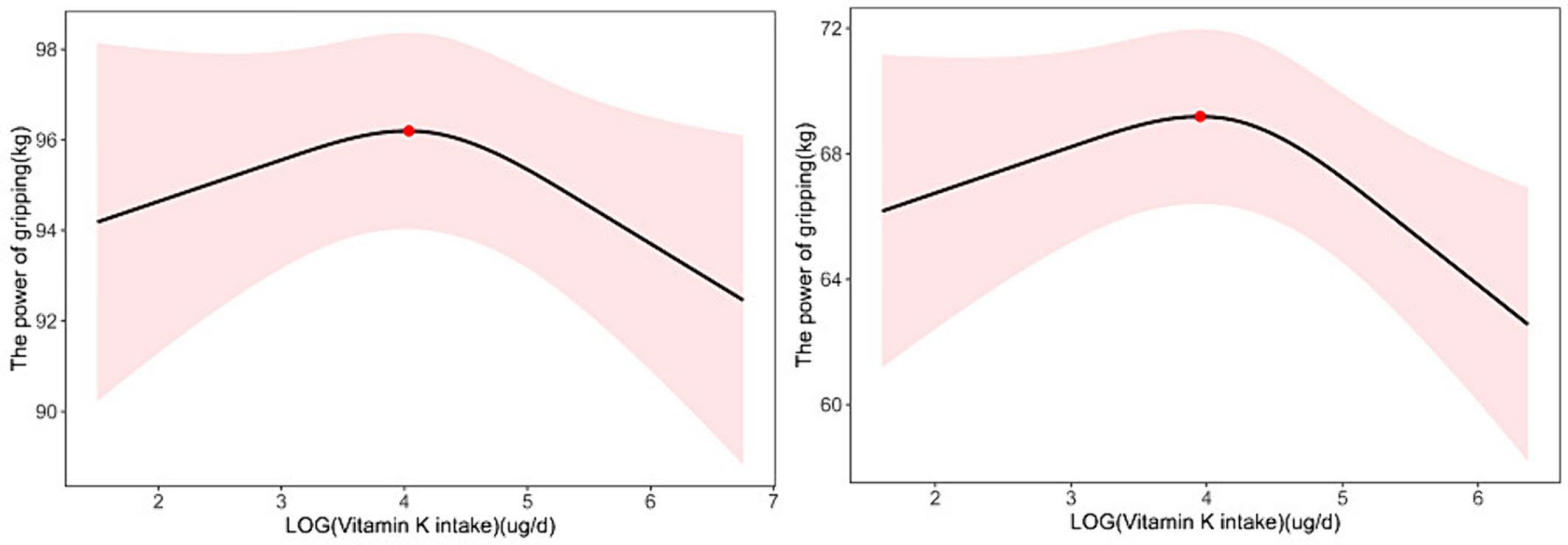
Nonlinear relationship between dietary vitamin K intake and handgrip strength in 18-44-year-old participants; the inflection point is 56.62808 μg/d (**A**). Nonlinear relationship between dietary vitamin K intake and handgrip strength in participants with BMI > 30; the inflection point is 52.049 μg/d (**B**).

In our multiple linear regression analysis, we used Model 2, which adjusted for all covariates except education, race, and protein intake due to collinearity issues (Supplementary Table S2). As shown in Table 1, we did not observe a linear association between dietary vitamin K intake and handgrip strength. However, we observed a similar N-shaped curve association between log-transformed dietary vitamin K intake and handgrip strength. The fitted curve is shown in Figure 2. The association between log-transformed dietary vitamin K intake and handgrip strength was positive before log-transformed dietary vitamin K intake, reaching approximately 4 (the turning point of the N-shaped curve), and then became negative. It appeared that there was a positive association between dietary vitamin K intake and handgrip strength in the range of 0–59.872 μg/d (*P* for nonlinearity =0.049). Increasing vitamin K intake within this range is associated with an improvement in handgrip strength. However, once dietary vitamin K intake exceeds 59.872 μg/d, handgrip strength does not continue to improve with further increases. Figure 3 also shows the median dietary vitamin K intake of this study population after logarithmic transformation (Median = 73.7 μg/d), which indicates that the dietary vitamin K intake of our study population is within an appropriate range.

### 3.4 Subgroup analysis

To determine whether the association between dietary vitamin K intake and ASMI and handgrip strength was stable in different subgroups, we performed subgroup analyses based on Model 2 grouped by age and BMI (Table 2; Figure 3). In adults aged 18–44 years, we found a linear relationship between dietary vitamin K intake and ASMI (β = 0.008, *p* = 0.021) and a nonlinear relationship between dietary vitamin K intake and handgrip strength (*P* for nonlinearity =0.048). When stratified by BMI, the results showed significant associations of vitamin K intake with ASMI and handgrip strength in overweight and obese participants, but the associations were not significant among lean participants (Table 2; Figure 3).

**Table 2:** Subgroup analysis of the association of dietary vitamin K intake with ASMI and handgrip strength.

	Linear		
Variable	β(95%CI)	*P*	Nonlinear
ASMI (kg/m^2^)			
Age			
18–44	0.008 (0.0018, 0.015)	0.021	0.856
45–59	0.000 (−0.013, 0.013)	0.993	0.056
BMI (kg/m^2^)			
<25	0.005 (−0.007, 0.017)	0.415	0.795
25–29.9	0.013 (0.003, 0.022)	0.012	0.780
≥30	0.003 (−0.005, 0.016)	0.592	0.215
Handgrip strength (kg)			
Age			
18–44	−0.011 (−0.075, 0.052)	0.707	0.048
45–59	0.003 (−0.012, 0.122)	0.964	0.367
BMI (kg/m^2^)			
<25	0.035 (−0.033, 0.103)	0.295	0.624
25–29.9	−0.010 (−0.101, 0.081)	0.822	0.138
≥30	−0.034 (−0.013, 0.065)	0.474	0.010

## 4 Discussion

The impact of skeletal muscle mass and strength loss on public health cannot be underestimated (38), as it is linked to higher morbidity and mortality rates, decreased quality of life, impaired physical functioning, and increased healthcare costs (39–41). Diet plays a critical role in maintaining muscle mass and strength, and previous research has highlighted the importance of various nutrients, including protein, amino acids, vitamin D, and magnesium (16). In recent years, there has been growing interest in the potential benefits of vitamin K for muscle metabolism, particularly based on results from laboratory experiments and animal models (42, 43). However, regarding studying its effects on human subjects through observational and interventional research, the results have been conflicting (25). While some studies suggest a positive association between dietary vitamin K intake and muscle health, others fail to find a significant relationship (43, 44).

To our knowledge, our study is the first to examine the associations between dietary vitamin K intake and skeletal muscle mass as well as muscle strength while appropriately identifying and adjusting for covariates. Previous clinical observational studies have yielded mixed results regarding the relationship between vitamin K status and physical performance (27, 44). However, these previous studies primarily focused on assessing plasma levels of vitamin K, whereas our research specifically focused on dietary vitamin K intake. By investigating the dietary aspect, our study provides valuable insights into the potential effects of vitamin K intake on muscle health, which has important implications for dietary recommendations and interventions.

In this large cross-sectional study of a representative population of adults from NHANES, our findings revealed a positive association between dietary vitamin K intake and skeletal muscle mass (as measured by ASMI) in males. Notably, sex differences were observed, suggesting potential underlying mechanisms for varying associations between dietary vitamin K intake and skeletal muscle mass. Previous studies indicated that men exhibit a higher rate of protein synthesis following similar stimuli, potentially due to elevated serum testosterone (45, 46). Serum testosterone reportedly increases muscle mass in a dose-dependent manner. Furthermore, evidence from animal experiments suggests that dietary vitamin K may impact testicular vitamin K levels, potentially resulting in the modulation of inflammation signal transduction and sustained testosterone levels (47, 48). Although they still need to be validated in humans, these insights might account for the observed sex disparities in the context of our study.

For muscle strength, the findings of this study revealed a significant positive association between dietary vitamin K intake and handgrip strength within the range of 0–59.872 μg/d. However, beyond this threshold (59.872 μg/d), increasing vitamin K intake did not lead to additional improvements in handgrip strength. This nonlinear relationship suggests that there is an optimal range of dietary vitamin K intake for maximizing muscle strength. Our study highlights the nonlinearity of the association between dietary vitamin K intake and muscle strength, providing valuable insights for optimizing vitamin K intake to enhance muscle strength. Excessive vitamin K supplementation beyond the identified threshold may not confer further benefits for muscle strength. Future research can focus on establishing evidence-based recommendations for optimal vitamin K intake to promote muscle health.

Subgroup analysis suggested a significant association between dietary vitamin K intake and muscle mass and strength among adults aged 18–44 years. Among overweight and obese participants, a significant association was observed between vitamin K intake and muscle mass and strength, which could be related to their chronic inflammatory status. This indicates that in subsequent researches, we could focus on elucidating the characteristics of vitamin K dietary intake, digestion, and absorption among individuals with abnormal body weight.

Our study presents a multitude of advantages. First, to guarantee integrity and accuracy, we used the proper weights and diligently adjusted for confounding variables during the analysis process. Second, it is noteworthy that previous studies investigating the correlation between vitamin K and muscle status have been limited in scale. In contrast, we conducted a comprehensive analysis utilizing a vast sample size obtained from the NHANES database, which provides robust and extensive data on a national level. Third, we clarified a nonlinear relationship and determined an inflection point, which provided the clinical value of our results. Last, this study included participants aged 18–59 years, which excluded the effect of age-related muscle disorders. By focusing on these individuals, we were able to achieve a more precise and accurate exploration of the relationship between vitamin K intake and muscle status.

However, there are some limitations in our study. First, importantly, due to the cross-sectional design of our study, we cannot establish a causal relationship between vitamin K intake and muscle status. Second, each participant’s dietary vitamin K intake was recorded through the first round 24-h dietary recall interview in our study. We have to admit that it is not able to accurately estimate usual dietary vitamin K intake, which could introduce the intra-individual variability. This is a limitation of using the NHANES database for dietary analysis. Third, there are three distinct forms of vitamin K: vitamin K1 (phylloquinone), K2 (menaquinone), and K3 (menadione). Within the diet, vitamin K primarily originates from two main sources: vitamin K1 and K2 (49). Vitamin K1 is abundant in vegetables and fermented foods, such as natto (50). Certain animal products, such as organ meat and certain cheeses, serve as rich sources of vitamin K2 (51, 52). Although diet is the major source of vitamin K, some VK metabolites are produced by the gut microbiota. Among nutritional supplements, vitamin K2 emerges as a potentially beneficial option. Despite the scarcity of studies directly examining the interrelationship between skeletal muscle and vitamin K2, existing evidence underscores the significant role of vitamin K2 in maintaining muscular homeostasis. The NHANES study does not distinguish between dietary vitamin K1 and vitamin K2, so the specific contributions of each subtype to our observed results cannot be differentiated. In subsequent research and clinical trials, our team will strive to distinguish the effects of different vitamin K subtypes on skeletal muscle, in order to obtain more accurate results.

## 5 Conclusion

In summary, we found a significant positive linear association between dietary vitamin K intake and skeletal muscle mass index in males. Our study also demonstrated a nonlinear relationship between dietary vitamin K intake and handgrip strength, highlighting an optimal intake range. Beyond a certain threshold, increasing vitamin K intake may not yield additional improvements in muscle strength.

## Data Availability

Publicly available datasets were analyzed in this study. This data can be found at: www.cdc.gov/nchs/nhanes/.
