# Supplementary material for: Dietary vitamin K intake in relation to skeletal muscle mass and strength among adults: a cross-sectional study based on NHANES
Source: Front Nutr. 2024 Aug 30;11:1378853. doi: 10.3389/fnut.2024.1378853 (PMC11392788; doi:10.3389/fnut.2024.1378853)
Supplement: Supplementary file 1 [file Data_Sheet_1.pdf]

Supplementary Material

1 Supplementary Tables

Supplemental Table 1. Multicollinearity in the covariates of the ASMI analysis

Variable	VIF	Variable	VIF	Variable	VIF
Vitamin K	2.581	marital	1.927	protein	14.471
Age	3.287	smoking	3.963	VD	2.399
Gender	2.659	High cholesterol	2.422	Glycohemoglobin	2.985
BMI		Edu	8.947	race	
25-29.9	2.742	high school/GED	10.662	Other Hispanic	2.382
>30	2.296	>high school	9.393	Non-Hispanic White	3.902
Mg	8.601	HDL	1.896	Non-Hispanic Black	5.026
hypertension	2.529	Total Cholesterol	2.831	Other Race	3.214
cancer	2.554	Ca	6.045	Dietary calories intake	13.715

Supplemental Table 2. Multicollinearity in the covariates of the handgrip strength analysis

Variable	VIF	Variable	VIF	Variable	VIF
Vitamin K	23.685	marital	5.973	protein	16.156
Age	8.744	smoking	24.910	VD	5.849
Gender	5.770	High cholesterol	8.748	Glycohemoglobin	12.751
BMI	46.176	Edu	48.567	race	99.390
Mg	11.651	HDL	4.016	Dietary calories intake	9.239
hypertension	13.194	Total Cholesterol	6.637		
cancer	7.810	Ca	11.513		

Supplemental Table 3. Characteristics of the study participants in the analysis of dietary vitamin K intake and skeletal muscle mas

Variable	All (11189)	Vitamin K				*P*
		Q1 (<41.0)	Q2(41.0-73.8)	Q3 (73.8-135.3)	Q4 (>135.3)	
Age						<0.001
18-44	7414 (63.9%)	1972 (69.3%)	1888 (65.1%)	1805 (62.6%)	1749 (59.3%)	
45-59	3775 (36.1%)	822 (30.7%)	905(34.9%)	999 (37.4%)	1049 (40.7%)	
Gender						<0.001
Male	5550 (50.4%)	1275 (45.7%)	1339(49.9%)	1501 (54.2%)	1435 (51.4%)	
Female	5639 (49.6%)	1519 (54.3%)	1454(50.1%)	1303 (45.8%)	1363 (48.6%)	
BMI[1]						<0.001
<25	3738 (32.9%)	881 (31.5%)	878(31.8%)	939 (31.9%)	1040 (36.2%)	
25-29.9	3443 (31.8%)	818 (28.9%)	844(29.3%)	886 (33.6%)	895 (35.1%)	
≥30	4004 (35.3%)	1092 (39.6%)	1070(39%)	979 (34.6%)	863 (28.7%)	
Race/ethnicity						<0.001
Mexican American	1718 (11.2%)	429 (11.53%)	482 (13.1%)	474 (11.78%)	333 (8.8%)	
Other Hispanic	1162 (7.4%)	310 (8.59%)	312(7.57%)	290 (7.2%)	250 (6.46%)	
Non-Hispanic White	3860 (59.6%)	960 (56.1%)	948(58.5%)	1006 (61.8%)	946 (61.4%)	
Non-Hispanic Black	2414 (11.8%)	709 (15.5%)	610 (11.8%)	553 (10.16%)	542 (10.22%)	
Other Race	2035 (10%)	386 (8.25%)	441 (9.1%)	481 (9.08%)	727 (13.1%)	
Smoking						<0.001
Every day	1854 (41.7%)	621 (55.1%)	478 (43.1%)	433 (38.2%)	332 (29.5%)	
Some days	517 (11.8%)	148 (12.0%)	117 (11.76%)	135 (11.68%)	117 (11.6%)	
Not at all	1722 (46.5%)	341 (32.9%)	417 (45.1%)	481 (50.1%)	483 (58.9%)	
Education						<0.001
<high school	616 (3.67%)	182 (4.52%)	186 (4.63%)	143 (3.3%)	105 (2.43%)	
high school/GED	4024 (32.8%)	1257 (43.1%)	1049 (34.7%)	962 (32.5%)	756 (22.6%)	
> high school	6236 (63.5%)	1270 (49.4%)	1481 (60.6%)	1642 (64.2%)	1870 (75%)	
Marital status						<0.001
Married or living with partners	6097 (60.4%)	1347 (54.9%)	1502 (61.1%)	1566 (59.4%)	1682 (65.2%)	
single	4160 (39.6%)	1132 (45.1%)	1040 (38.9%)	1034 (40.6%)	954 (34.8%)	

Hypertension						0.658
Yes	2460 (21.5%)	619 (21.3%)	610 (22.9%)	645 (20.9%)	586 (21.2%)	
No	8719 (78.5%)	2171 (78.7%)	2183 (77.1%)	2154 (79.1%)	2211 (78.8%)	
High Cholesterol						0.010
Yes	2526 (24.4%)	556 (21.5%)	613 (23.5%)	664 (25%)	693 (27.3%)	
No	8378 (75.6%)	2139 (78.5%)	2097 (76.5%)	2079 (75%)	2063 (72.7%)	
Cancer						0.158
YES	393 (5.09%)	98 (5.43%)	90 (4.3%)	94 (4.38%)	111 (6.23%)	
NO	9862 (94.01%)	2381 (94.6%)	2452 (95.7%)	2504 (95.6%)	2525 (93.8%)	
Protein[2]	86.25±0.63	62.41±1.25	81.75±1.15	94.78±1.27	102.64±1.51	<0.001
Ca[3]	1080.8±11.29	803.62±18.66	1051.4±16.86	1188.1±20.35	1242.4±17.44	<0.001
Mg[4]	331.36±3.08	227.14±4.67	299.82±3.96	359.38±5.28	423.01±5.46	<0.001
Vitamin D[5]	14.03±0.71	9.80±1.32	11.73±1.08	14.62±1.56	19.21±1.61	<0.001
HDL[6]	1.36±0.01	1.32±0.01	1.36±0.02	1.35±0.01	1.42±0.01	<0.001
Total_Cholesterol[7]	4.93±0.02	4.93±0.03	4.90±0.03	4.94±0.03	4.96±0.03	0.203
Glycohemoglobin[8]	5.5±0.01	5.5±0.02	5.51±0.02	5.51±0.02	5.48±0.02	0.712
Dietary calories intake[9]	2239.5±11.16	1655.9±21.68	2153.3±20.48	2471.1±26.22	2596.5±31.67	<0.001

[1] Body Mass Index (kg/m^2).
[2] Dietary protein intake (gm).
[3] Dietary Calcium intake (mg).
[4] Dietary Magnesium intake (mg).
5 Dietary Vitamin D intake (ug/d).
[6] HDL-Cholesterol (mmol/L).
[7] Total Cholesterol(mmol/L).
[8] Glycohemoglobin (%).
[9] Dietary calories intake (kcal).

Supplemental Table 4. Characteristics of the study participants in the analysis of dietary vitamin K intake and muscle strengt

Variable	All (6892)	Vitamin K				P
		Q1 (<41.5)	Q2(41.5-73.7)	Q3 (73.7-133.8)	Q4 (>133.8)	
Age						<0.001
18-44	4622 (63.4%)	1229 (69.6%)	1185 (64.7%)	1119 (62.1%)	1089 (58.2%)	
45-59	2270 (36.6%)	489 (30.4%)	542 (35.3%)	603 (37.9%)	636 (41.8%)	
Gender						0.003
Male	3404 (50.5%)	765 (44.6%)	814 (49.6%)	927 (53.8%)	898 (53%)	
Female	3488 (49.5%)	953 (55.4%)	913 (50.4%)	795 (46.2%)	827 (47%)	

BMI[1]						0.021
<25	2326 (33.3%)	546 (32.3%)	548 (31.8%)	589 (32.9%)	643 (35.8%)	
25-29.9	2074 (31.6%)	497 (29.1%)	504 (29.6%)	536 (33.3%)	537 (33.9%)	
≥30	2448 (35.2%)	655 (38.6%)	670 (38.6%)	584 (33.9%)	539 (30.3%)	
Race/ethnicity						0.004
Mexican American	890 (10.3%)	209 (9.3%)	262 (13.4%)	240 (10.7%)	179 (7.8%)	
Other Hispanic	618(6.3%)	163 (7.8%)	167 (6.6%)	151 (5.7%)	137 (5.3%)	
Non-Hispanic White	2628(66.2%)	650 (58.4%)	636 (59.4%)	701 (65.1%)	641 (65.3%)	
Non-Hispanic Black	1611(12.6%)	470 (16.3%)	417 (12.8%)	354 (10.6%)	370 (11.2%)	
Other Race	1145 (8.6%)	226 (8.2%)	245 (7.8%)	276 (7.9%)	398 (10.4%)	
Smoking						<0.001
Every day	1248 (44.4%)	409 (58.4%)	318 (45.4%)	315 (44.0%)	206 (29.7%)	
Some days	297(11.0%)	78 (10.6%)%)	72 (10.9%)	72 (10.2%)	75 (12.4%)	
Not at all	1046 (44.6%)	200 (31.0%)	260 (43.7%)	282 (45.8%)	304 (57.9%)	
Education						<0.001
<high school	312(3.3%)	92 4.34%)	96 (4.3%)	74 (2.9%)	50 (2.1%)	
high school/GED	2486(33.1%)	753 (40.9%)	649 (34.8%)	601 (33.8%)	483 (24.1%)	
> high school	3903(63.6%)	816 (54.7%)	941 (60.9%)	1008 (63.3%)	1138 (73.8%)	
Marital status						0.044
Married or living with partners	3662 (59.4%)	831 (55.3%)	911 (60.1%)	932 (60.0%)	988 (61.6%)	
single	2667 (40.6%)	700 (44.7%)	677 (39.9%)	649 (40.0%)	641 (38.4%)	
Hypertension						0.930
Yes	1569 (22.9%)	396 (22.2%)	392 (23%)	416 (23.2%)	365 (23.1%)	
No	5317 (77.1%)	1320 (77.8%)	1334 (77%)	1304 (76.8%)	1359 (73.9%)	
High Cholesterol						0.058
Yes	1607(26.5%)	358 (22.6%)	384 (25.8%)	437 (28.9%)	428 (28.0%)	
No	4985(73.5%)	1253 (77.4%)	1263 (74.2%)	1222 (71.1%)	1247 (72.0%)	
Cancer						0.591
YES	251 (5.4%)	60 (5.3%)	58 (4.7%)	55 (5.1%)	78 (6.5%)	
NO	6077 (94.6%)	1471 (94.7%)	1529 (95.4%)	1526 (94.0%)	1551 (93.5%)	
Protein[2]	87.33±0.67	63.75±1.76	82.44±1.26	95.59±1.39	103.83±2.15	<0.001
Ca[3]	1120.7±15.93	822.64±28.45	1077.5±20.11	1239.5±25.28	1297.6±24.13	<0.001
Mg[4]	334.02±4.23	231.9±7.65	302.99±5.45	364.38±5.98	420.22±6.62	<0.001

Vitamin D intake[5]	12.509±0.82	6.86±0.59	12.48±1.65	12.54±0.60	17.36±2.16	<0.001
HDL[6]	1.35±0.01	1.32±0.02	1.34±0.02	1.33±0.02	1.40±0.01	<0.001
Total_Cholesterol[7]	4.97±0.03	4.96±0.04	4.91±0.04	5.03±0.04	4.97±0.04	0.0323
Glycohemoglobin[8]	5.49±0.01	5.44±0.02	5.49±0.03	5.54±0.03	5.47±0.02	0.1224
Dietary calories intake[9]	2276.5±13.30	1697±27.58	2149±19.47	2525.8±34.07	2642.4±41.00	<0.001

[1] Body Mass Index (kg/m^2).
[2] Dietary protein intake (gm).
[3] Dietary Calcium intake (mg).
[4] Dietary Magnesium intake (mg).
5 Dietary Vitamin D intake (ug/d).
[6] HDL-Cholesterol (mmol/L).
[7] Total Cholesterol(mmol/L).
[8] Glycohemoglobin (%).
[9] Dietary calories intake (kcal).

2 Supplementary Figures

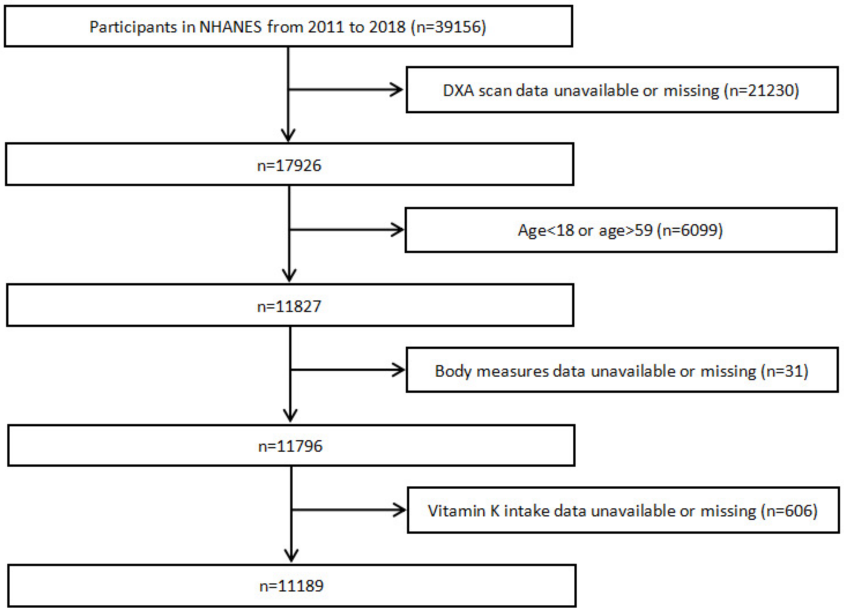

Supplementary Figure 1. Flow chart of participants selection in the study of dietary vitamin K intake and skeletal muscle mass

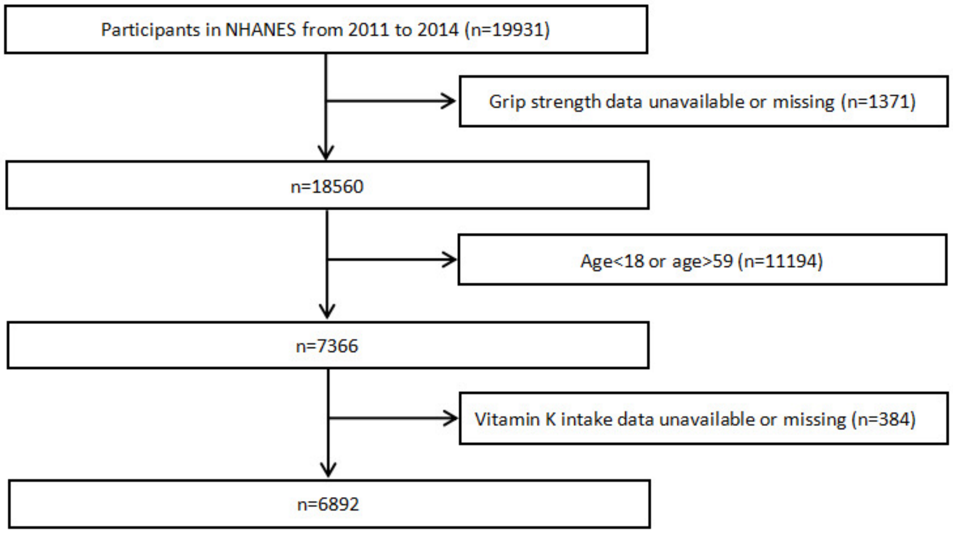

Supplementary Figure 2. Flow chart of participants selection in the study of dietary vitamin K intake and muscle strength
